# Adjustment of Prognostic Effects in Prevalent Case-control Studies on Genotype

**DOI:** 10.2188/jea.11.204

**Published:** 2007-11-30

**Authors:** Nobuyuki Hamajima, Keitaro Matsuo, Hidemichi Yuasa

**Affiliations:** 1 Division of Epidemiology and Prevention, Aichi Cancer Center Research Institute.; 2 Nagoya University Graduate School of Medicine.; 3 Department of Oral and Maxillofacial Surgery, Nagoya City Jyouhoku Municipal Hospital.

**Keywords:** prevalent case-control study, prognostic effect, genetic polymorphism

## Abstract

Since genotypes are unchangeable, adjustment of prognostic effects in prevalent case-control studies may produce an unbiased estimate of odds ratio (OR) for disease occurrence. In this paper, the prognostic effects on OR is demonstrated, then three approaches to examine and/or adjust the OR are presented. The demonstration shows that the prognostic effects are larger in diseases with poor prognosis than in those with better prognosis. Genotypes increasing disease risk and fatality rate are underestimated, while those increasing the risk and improving prognosis are overestimated. The simplest approach to examine the OR derived from prevalent case-control studies is to conduct stratified analysis according to the interval between diagnosis and study enrollment. When the stratified analysis finds no substantial difference in the estimate, the OR reflects mainly the relative risk for disease occurrence. The proportion of genotype among putative cases at diagnosis can be estimated from prevalent cases by a logistic model, producing the OR adjusted for the interval from diagnosis. An incomplete-data case-control design is also applicable to adjust the prognostic effects. An actual prevalent case-control study on breast cancer is used to demonstrate the three approaches. They are useful to compensate the disadvantage of prevalent case-control studies.

## INTRODUCTION

Case-control studies with prevalent cases for estimating a relative risk have been regarded as a substandard design due to two reasons. One is that an estimated odds ratio (OR) of factors under study indicates an association not only with disease occurrence but also with disease prognosis^1^^-^^3^^)^. In prevalent case-control studies, longer-term survivors are more likely to be sampled as cases. Accordingly, the factors related to longer survival are more frequent among prevalent cases than among incident cases, which produces an elevated OR even if the frequency among incident cases is the same among controls. Such prognostic effects introduce a bias for the OR estimation concerning relative disease occurrence. The other relates to the recall bias. The memory concerning exposure experience before disease onset becomes more obscure as the time interval between diagnosis and enrollment is larger, so that the difference in the accuracy of exposure status between cases and controls is larger in prevalent case-control studies than in incident case-control studies. This uncorrectable recall bias has been discouraging to develop a method to adjust the prognostic effects in prevalent case-control studies.

Along with a rapid progress of genotyping techniques using PCR (polymerase chain reaction)^4^^-^^7^^)^, many epidemiologic studies have been conducted to estimate the relative risk of genetic polymorphisms^8^^-^^11^^)^, by case-control studies with incident or prevalent cases. Since the genotypes do not change and are independent on the time of genotype tests, no information bias is included for the estimation of genotype OR. Accordingly, if a model adjusting the prognostic effects is introduced, the disadvantages of prevalent case-control studies will be overcome, resulting in a comparable design to incident case-control studies.

This paper proposes approaches to examine and/or adjust the prognostic effects in prevalent case-controls studies on genotypes. First, prognostic effects on the estimated ORs are simulated by a mathematical model, followed by three approaches; 1) stratified analysis according to the time interval between diagnosis and study enrollment, 2) estimation of the percentage of individuals with a given genotype among putative cases at diagnosis (putative incident cases), and 3) a Poisson regression model to adjust the OR for the interval.

A prevalent case-control study on breast cancer risk and *beta 2 adrenergic receptor* gene (*BAR2*) *Gln27Glu* polymorphism^12^^)^ is used as an example to apply the approaches. It is known that adrenergic receptors play roles in the regulation of thermogenesis and lipid mobilization. The *Glu* allele of *BAR2 Gln27Glu* polymorphism was reported to be associated with obesity^13^^)^, though inconsistent results were also reported^14^^)^. Since obesity is a risk factor for postmenopausal women, we conducted the case-control study.

## MATERIALS AND METHODS

### Study subjects

The breast cancer case-control study used as an example was conducted in a series of projects^15^^, ^^16^^)^ approved by the Ethical Committee at Aichi Cancer Center in 1999 (Ethical Committee Approval Numbers 12-20 and 12-23). Cases were 239 female breast cancer patients aged 26 to 70 years (mean, 50.4 years) at diagnosis, who had been diagnosed in the past 20 years at Aichi Cancer Center Hospital. Controls were 186 female outpatients aged 24 to 69 years (mean, 53.0 years) without cancer who visited the same hospital, mainly at clinics of gastroenterology, breast surgery, and gynecology. All subjects were enrolled between 1999 and 2000.

Their *BAR2 Gln27Glu* polymorphism was genotyped by the method described by Large et al^13^^)^. The subjects with a *Glu* allele (*GlnGlu* or *GluGlu* genotype) were 30 (12.6%) out of 239 cases, and 32 (17.2%) out of 186 controls.

### Statistical models

#### 1. Prognostic effects on OR

In order to examine the prognostic effects on OR, a mathematical model is constructed. The OR for a given genotype is calculated by

$$OR=\{p_{\text{case}}(1-p_{\text{control}})\}/\{(1-p_{\text{case}})p_{\text{control}}\},$$

where p_contro_l is the proportion of the genotype among controls and p_case_ is among cases. When OR is given, p_case_ is obtained by

$$p_{\text{case}}=\{OR\;p_{\text{control}}\}/\{1+p_{\text{control}}(OR-1)\}.$$

Denote the survival curve for those without the genotype as S(*t*), *a* as a constant, and *t* as the time from diagnosis, and assume S(*t*) to be expressed by

S(t)=exp⁡(−at).

When hazard ratio (HR) for those with the genotype relative to those without it is given, the survival curve for those with the genotype, S(*t*)*_g_*, is

$$S(t{)}_{\text{g}}=\exp(-a*HR*t).$$

Accordingly, the proportion of those with the genotype among the survivors at time *t*, which is denoted by p_g_, is expressed by

$$p_{\text{g}}=p_{\text{case}}\;S(t{)}_{\text{g}}/\{p_{\text{case}}\;S(t{)}_{\text{g}}+(1-p_{\text{case}})S(t)\}.$$

The OR′ derived from the case-control study with prevalent cases at time *t* is calculated by p_control_ and p_g_, as following.

$${OR}^{\prime }=p_{\text{g}}(1-p_{\text{control}})/\{(1-p_{\text{g}})p_{\text{control}}\}.$$

#### 2. Stratified analysis according to the interval from diagnosis

Stratified analysis is an easy method to examine the prognosis effects on OR. The cases with a specified range of the interval between diagnosis and study enrollment are used for estimating their OR in an ordinary method. When the estimated OR for the cases with a longer interval is similar to that for the cases with a shorter interval, the OR indicates that the effect is mainly due to the association with disease occurrence. The example data on the intervals between diagnosis and study enrollment are shown in Table 1.

**Table 1.** Intervals between diagnosis and study enrollment of breast cancer patients in example prevalent case-control study.

	*GlnGln* genotype		*GlnGlu/GluGlu* genotype	
	
	*n*	%	*n*	%
< 1 year	69	33.0	10	33.3
≥ 1 and < 2 years	29	13.9	4	13.3
≥ 2 and < 3 years	25	12.0	3	10.0
≥ 3 and < 4 years	32	15.3	2	6.7
≥ 4 and < 5 years	23	11.0	6	20.0
≥ 5 and < 6 years	22	10.5	3	10.0
≥ 6 and < 7 years	2	1.0	0	0.0
≥ 7 and < 8 years	1	0.5	0	0.0
≥ 8 and < 9 years	2	1.0	1	3.3
≥ 9 and < 10 years	0	0.0	0	0.0
≥ 10 and < 11 years	2	1.0	0	0.0
≥ 11 and < 12 years	0	0.0	1	3.3
≥ 12 and < 17 years	0	0.0	0	0.0
≥ 17 and< 18 years	1	0.5	0	0.0
≥ 18 and < 20 years	0	0.0	0	0.0
≥ 20 and < 21 years	1	0.5	0	0.0

Total	209	100	30	100

#### 3. Genotype proportion adjustment approach

If the proportion of individuals with a given genotype among putative incident cases can be estimated for given prevalent cases, we can calculate the OR for disease risk using the estimated proportion. A logistic regression model may provide the proportion based on the interval distribution of the cases according to the genotype. When the interval from diagnosis is denoted as *t*, and p*_t_* as the proportion of cases with the genotype at *t*, the model is expressed by

$$\log\{\{p_{t}/(1-p_{t})\}=\alpha +\beta t,$$

where *α* and *β* are coefficients of the model. The data on *t* and genotype (1 for having the genotype and 0 for not having) for each case are analyzed by the statistical package, providing the estimates for *α* and *β*. The proportion of the genotype among the putative incident cases is p*_t_* at *t* = 0, denoted as p_incident_. It is calculated by

$$p_{\text{incident}}=\exp(\alpha )/\{1+\exp(\alpha )\}.$$

The OR adjusted for the interval from diagnosis, aOR, is calculated by

$$aOR=\{p_{\text{incident}}(1-p_{\text{control}})\}/\{(1-p_{\text{incident}})p_{\text{control}}\}.$$

#### 4. Incomplete-data case-control design approach

A Poisson regression model has been fully described for estimating gene-environment interaction in incomplete-data case-control design^17^^)^, where the data on environmental exposure (or genotype) are not available for controls. Table 2 shows the data structure for a prevalent case-control study we propose in this paper, as well as for a gene-environment interaction study^17^^)^. Without the exposure data according to genotype among controls, the model provides the unbiased OR for genotype, as well as the estimate for interaction term, under the assumption that there is no association between the distributions of genotype and exposure among controls.

**Table 2.** Incomplete-data case-control design for gene-environment interaction study (exposure) and for prevalent case-control study (interval).

		Controls			Cases			
	
		Exposure			Exposure		Interval	
		No	Yes	Total	No	Yes	Short	Long
Genotype	No	UK*	UK	U_00*_	U_100_	U_101_	U_100_	U_101_
	Yes	UK	UK	U_01*_	U_110_	U_111_	U_110_	U_111_

Total		UK	UK	U_0**_	U_1*0_	U_1*1_	U_1*0_	U_1*1_

* : Unknown

Since the concept of the interval between diagnosis and study enrollment is not applicable for controls, no association between the interval and genotype distribution among controls could be regarded in the model. Accordingly, the incomplete-data case-control design can be similarly applicable for prevalent case-control studies. The model is expressed by

$$\log(u_{\text{dgt}})=\mu_{0}+\alpha_{0}G+\alpha_{1}DT+\beta_{1}DG+\gamma_{1}DGT,$$

where u_dgt_ denotes the expected number of subjects, D disease status (0 for controls and 1 for cases), G genotype (0 for baseline genotype and 1 for target genotype), and T interval from diagnosis (0 for a shorter interval and 1 for a longer interval). In this model, the log(*β*_1_) provides the estimates of the OR adjusted for the prognostic effects, which can be interpreted as the relative risk of the genotype for disease occurrence.

### Calculations

The above estimates were calculated by the computer program STATA Version 7 (STATA Corporation, College Station, TX). The ORs and 95% confidence intervals (95%CI) were estimated by STATA commands “logistic” and “logit” for stratified analysis according to the interval from diagnosis, “cci” for genotype proportion adjustment approach, and “poisson” for incomplete-data case-control design approach.

## RESULTS

### 1. Prognostic effects on ORs

We assume here that all prevalent cases are the survivors at time t with S(*t*). It is an extreme case, because prevalent case-control studies usually include a proportion of incident cases as well as prevalent cases with a different *t*. Table 3 shows the calculated OR′ when p_control_ = 0.1, 0.3, or 0.5, OR=2 or 5, S(*t*)=0.25, 0.5, or 0.75, and HR=0.5, 1, or 2. In case of HR=1, i.e., the genotype is not influential to prognosis, the same estimate is obtained as the true OR. When the genotype relates to poor prognosis (HR>1), the OR′s become small. Meanwhile, when it relates to better prognosis (HR<1), the OR′s become large. When the survival rate is lower, the effect on the ORs is larger. When the survivors are half and their HR is 2, OR=2 is reduced to OR′=1 and OR=5 to OR′=2.5. In case of S(*t*)=0.75, the effect is relatively small; OR=2 is reduced to OR′=1.5 for HR=2 and increased to OR′=2.3 for HR=0.5. The OR′ for OR=5 with S(*t*)=0.75 is 3.8 for HR=2 and 5.8 for HR=0.5. The extent is independent on p_control_, the proportion of the controls having the genotype.

**Table 3.** Calculated OR′ for prevalent case-control study according to S(*t*) and HR.

P_control_	S(*t*)	HR	S(*t*)*_g_*	OR=2			OR=5		
	
				p_case_	p*_g_*	OR′	p_case_	p*_g_*	OR′
0.1	0.25	0.5	0.500	0.182	0.308	4.000	0.357	0.526	10.000
		1	0.250	0.182	0.182	2.000	0.357	0.357	5.000
		2	0.063	0.182	0.053	0.500	0.357	0.122	1.250
	0.5	0.5	0.707	0.182	0.239	2.838	0.357	0.440	7.071
		1	0.500	0.182	0.182	2.000	0.357	0.357	5.000
		2	0.250	0.182	0.100	1.000	0.357	0.217	2.500
	0.75	0.5	0.866	0.182	0.204	2.309	0.357	0.391	5.774
		1	0.750	0.182	0.182	2.000	0.357	0.357	5.000
		2	0.563	0.182	0.143	1.500	0.357	0.294	3.750

0.3	0.25	0.5	0.500	0.462	0.632	4.000	0.682	0.811	10.000
		1	0.250	0.462	0.462	2.000	0.682	0.682	5.000
		2	0.063	0.462	0.176	0.500	0.682	0.349	1.250
	0.5	0.5	0.707	0.462	0.548	2.828	0.682	0.752	7.071
		1	0.500	0.462	0.462	2.000	0.682	0.682	5.000
		2	0.250	0.462	0.300	1.000	0.682	0.517	2.500
	0.75	0.5	0.866	0.462	0.497	2.309	0.682	0.712	5.774
		1	0.750	0.462	0.462	2.000	0.682	0.682	5.000
		2	0.563	0.462	0.391	1.500	0.682	0.616	3.570

0.5	0.25	0.5	0.500	0.667	0.800	4.000	0.833	0.909	10.000
		1	0.250	0.667	0.667	2.000	0.833	0.833	5.000
		2	0.063	0.667	0.333	0.500	0.833	0.555	1.250
	0.5	0.5	0.707	0.667	0.739	2.828	0.833	0.876	7.071
		1	0.500	0.667	0.667	2.000	0.833	0.833	5.000
		2	0.250	0.667	0.500	1.000	0.833	0.714	2.500
	0.75	0.5	0.866	0.667	0.698	2.309	0.833	0.852	5.774
		1	0.750	0.667	0.667	2.000	0.833	0.833	5.000
		2	0.563	0.667	0.600	1.500	0.833	0.789	3.570

### 2. Stratified analysis

Table 4 shows the ORs of breast cancer derived from the example case-control study. The overall OR is 0.691 (95%CI, 0.403-1.185). The OR for the cases less than one year after diagnosis is 0.697 (0.325-1.498). When the cases are divided into two groups (less than 2 years vs. 2 years or more), the first two digits of the estimates are the same; OR=0.69. Although the cases five years or over after diagnosis shows a larger OR than the other estimates.

**Table 4.** Odds ratios (ORs) and 95% confidence interval (95%CI) according to the interval from diagnosis.

	Cases			Controls	OR	(95%CI)
	
	N^a^	*Glu* ^b^	*Glu*/N^c^	N		
< 1 year	79	10	0.127	186	0.697	(0.325-1.498)
< 2 years	112	14	0.125	186	0.688	(0.345-1.353)
≥ 2 years	127	16	0.126	186	0.694	(0.363-1.326)
≥ 2 and < 5 years	91	11	0.121	186	0.662	(0.317-1.382)
≥ 5 years	36	5	0.139	186	0.776	(0.280-2.149)

All cases	239	30	0.126	186	0.691	(0.403-1.185)

a: Number of cases.

b: *Glu* allele carriers of *beta 2 adrenergic receptor* gene.

c: the proportion of *Glu* allele carriers.

### 3. Adjusted genotype proportion approach

**Figure 1.**
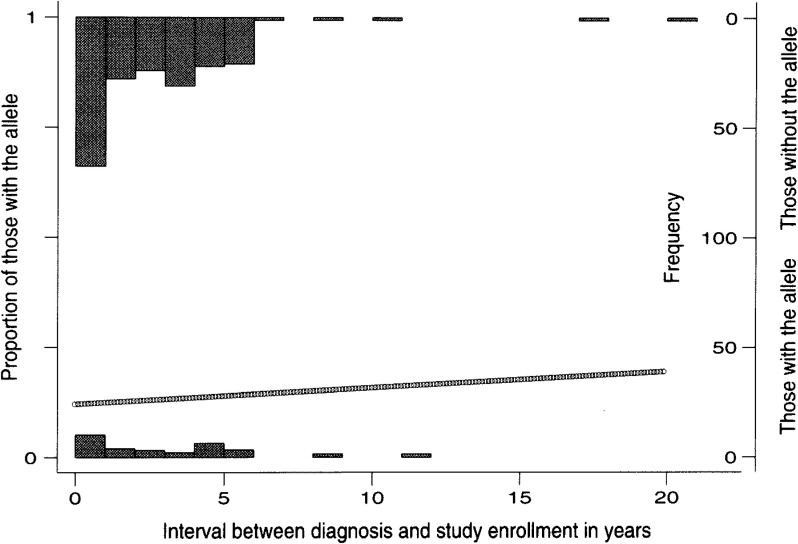
Estimation of genotype proportion of putative incident cases at diagnosis for a prevalent case-controls study on breast cancer and *beta-2 adrenergic receptor Glu* allele by a logistic model.

The proportion of putative cases with the genotype sampled at diagnosis, p_incident_, is needed for estimating the OR. The logistic model applied for the sample data on Table 1 provides *α*=-2.0124 and *β*=0.0303. The logistic curve is depicted in Figure 1. The p_incident_ is calculated to be 0.118 from the value of *α*. The corresponding proportion among all cases was 0.126 (30/239), which is slightly higher than the estimated p_incident_. The OR is 0.691 for unadjusted and 0.644 for adjusted.

As shown in Table 4, the proportion of *Glu* allele carriers is stable for the cases less than 5 years from diagnosis. When they (n=203) are applied for the estimation of p_incident_, *α*=2.0320 and *β*=0.0446 are obtained, giving p_incident_=0.116 and OR=0.632.

### 4. Incomplete-data case-control design approach

Table 5 shows the adjusted ORs when the interval is categorized into two periods (<1 year vs. ≥1 year, and <2 years vs. ≥2 years). There is no substantial difference in the OR between the two different cut points; both are around 0.69 with a wider confidence interval (0.325-1.498 and 0.349-1.353, respectively) than the unadjusted estimated (0.404-1.118). The estimates are close to those obtained by stratified analysis and genotype proportion adjustment approach.

**Table 5.** Incomplete-data case-control design approach to the OR estimation adjusted for the interval after diagnosis by a Poisson regression model.

Genotype	Controls	Cases			

		Interval from diagnosis			
		< 1 year	≥ 1 year	< 2 year	≥ 2 year
*GlnGln*	154	60	140	98	111
*GlnGlu/GluGlu*	32	10	20	14	16

Total	186	79	160	112	127

aOR		0.697	(0.325-1.498)	0.688	(0.349-1.353)

## DISCUSSION

Although prevalent case-control studies have two main problems as stated in the introduction, they are very attractive because of a shorter enrollment period of study subjects. A short enrollment period suits well, especially in rapidly changing research fields. In addition, a larger number of subjects sampled from prevalent cases produces a more stable estimate than limited number of incident cases collected in the same length of enrollment period. Epidemiologists have been considering prevalent case-control studies to be a substandard, unrecommendable design, because their main tool in the past was questionnaires. Questionnaire studies are sometimes very cheap and powerful, but in many cases information bias is inevitable. For information-bias-free risk factors, the adjustment of the prognostic effects enables us to produce evidence at a similar level to that from incident case-control studies. Modem biostatistical models and computer programs provide us rough, but simple adjustment approaches to examine and adjust the ORs from prevalent case-control studies. Even if they are not accurate in a statistical sense, these approaches are apparently useful. Until a more sophisticated method is developed, they could be applied for prevalent case-control studies, producing new findings.

Whether environmental exposure or genetic traits, factors affecting both disease occurrence and prognosis may work in the same direction; either to promote or disturb a disease process. Smoking elevates disease risk, and for many diseases it deteriorates the prognosis^18^^, ^^19^^)^. In such a case, the observed OR (i.e., OR′) is the underestimated, indicating that the effect on disease occurrence is larger than the observed one. For the prevalent cases with a 50% of survival rate on average, the OR′ is unity for OR=2 and HR=2. It means that OR larger than HR stays the same side (>1 or <1) for the cases with more than 50% survivorship. Another important feature is that a large OR for the cases with a large survival rate does not affect the conclusion of the study. The OR′ for OR=5 with S(*t*)=0.75 in Table 3 is 3.8 for HR=2 and 5.8 for HR=0.5. It may be a smaller deviation than a random deviation from a small size study, and than the difference among the different studies.

The simplest method to examine the prognostic effects is the stratified analysis. If the ORs obtained by the stratified analysis are more than unity or less than unity, the factor may be associated with the disease occurrence. When no substantial difference is observed among the ORs, the prognostic effects on the observed OR is limited.

If the prognostic effects of genotype under study cannot be neglected, the adjustment is recommended. Although this paper did not compare the genotype proportion adjustment approach with the incomplete-data case-control design approach in statistical viewpoints, the choice may be dependent on the distribution of the interval from diagnosis and function of the genotype ratio according to the interval. The conditions causing a large difference in the adjusted ORs between the two approaches remain to be elucidated.

The example data in this study is from a prevalent case-control study of breast cancer, which has a good prognosis. Probably, the survival rate is 0.8 or more on average for the present cases whose intervals from diagnosis are distributed mainly between 0 and 5 years. Accordingly, the difference among the estimates is not observed. The application to the cases with poor prognosis could show a clearer adjustment effect, as expected from Table 1. To date, we do not have such an actual dataset.

Genetic epidemiology opened a new era in the field of epidemiology. It seems a dramatic change similar to one at a half century ago from infectious disease epidemiology to modem epidemiology. Host factors have been discussed conceptually among the epidemiologists for decades, but now we can investigate host factors in a concrete form by genotyping techniques. Together with the shift from solely environmental factor evaluation to interactions with genetic traits, new methodology has been emerged^17^^, ^^20^^-^^23^^)^. An incomplete-data case-control design under the assumption of the independent distributions of exposure and genotype seems a powerful epidemiologic tool. The method is demonstrated in this paper to be applicable for the adjustment of the prognostic effects. All three approaches are straightforward and understandable, which could compensate the disadvantages of prevalent case-control studies.
